# Classical BSE prions emerge from asymptomatic pigs challenged with atypical/Nor98 scrapie

**DOI:** 10.1038/s41598-021-96818-2

**Published:** 2021-08-31

**Authors:** Belén Marín, Alicia Otero, Séverine Lugan, Juan Carlos Espinosa, Alba Marín-Moreno, Enric Vidal, Carlos Hedman, Antonio Romero, Martí Pumarola, Juan J. Badiola, Juan María Torres, Olivier Andréoletti, Rosa Bolea

**Affiliations:** 1 Centro de Encefalopatías y Enfermedades Transmisibles Emergentes, Universidad de Zaragoza IA2 IIS Aragón, C/ Miguel Servet 177, 50013 Zaragoza, Spain; 2 UMR Institut National de La Recherche Agronomique (INRA)/École Nationale Vétérinaire de Toulouse (ENVT) 1225, Interactions Hôtes Agents Pathogènes, 31076 Toulouse, France; 3 Centro de Investigación en Sanidad Animal (CISA-INIA), 28130 Valdeolmos, Madrid Spain; 4 Centre de Recerca en Sanitat Animal, Universitat Autònoma de Barcelona (UAB)-Institut de Recerca i Tecnologia Agroalimentàries, 08193 Bellaterra, Barcelona Spain; 5 Servicio de Cirugía y Medicina Equina, Hospital Veterinario, Universidad de Zaragoza, 50013 Zaragoza, Spain; 6 Departament de Medicina i Cirurgia Animals, Facultad de Veterinaria, Universitat Autònoma de Barcelona (UAB), 08193 Bellaterra, Barcelona Spain

**Keywords:** Diseases of the nervous system, Infectious diseases

## Abstract

Pigs are susceptible to infection with the classical bovine spongiform encephalopathy (C-BSE) agent following experimental inoculation, and PrP^Sc^ accumulation was detected in porcine tissues after the inoculation of certain scrapie and chronic wasting disease isolates. However, a robust transmission barrier has been described in this species and, although they were exposed to C-BSE agent in many European countries, no cases of natural transmissible spongiform encephalopathies (TSE) infections have been reported in pigs. Transmission of atypical scrapie to bovinized mice resulted in the emergence of C-BSE prions. Here, we conducted a study to determine if pigs are susceptible to atypical scrapie. To this end, 12, 8–9-month-old minipigs were intracerebrally inoculated with two atypical scrapie sources. Animals were euthanized between 22- and 72-months post inoculation without clinical signs of TSE. All pigs tested negative for PrP^Sc^ accumulation by enzyme immunoassay, immunohistochemistry, western blotting and bioassay in porcine PrP mice. Surprisingly, in vitro protein misfolding cyclic amplification demonstrated the presence of C-BSE prions in different brain areas from seven pigs inoculated with both atypical scrapie isolates. Our results suggest that pigs exposed to atypical scrapie prions could become a reservoir for C-BSE and corroborate that C-BSE prions emerge during interspecies passage of atypical scrapie.

## Introduction

Transmissible spongiform encephalopathies (TSE) are neurodegenerative diseases that affect humans and various animal species and include scrapie (classical and atypical) in sheep and goats, chronic wasting disease (CWD) in cervids, bovine spongiform encephalopathy (BSE) in cattle and variant Creutzfeldt-Jakob disease (vCJD) in humans. They are characterized by the presence of a misfolded and neurotoxic protein (PrP^Sc^), an abnormal isoform of a glycoprotein encoded by the host called cellular PrP (PrP^C^). Both proteins share the same amino acid sequence, but differ in their secondary structure^1^. The natural transmission of TSE in animals usually involves an oral exposure to the agent^2^.

In the pigs, to date, no field cases of TSE of natural origin have been described, despite the fact that epidemiological studies performed in the United Kingdom showed that they were exposed to contaminated feed containing ruminant meat and bone meal^3,4^. However, pigs are susceptible to parenteral experimental infection with classical BSE (C-BSE), and sheep BSE (SBSE) agents^4–7^. We previously demonstrated that pigs are efficiently infected with the SBSE agent, which leads to a 100% attack rate when inoculated intracerebrally in pigs and a widespread distribution of PrP^Sc^, which is present in a wide variety of peripheral tissues and blood components from these animals^7,8^. However, SBSE has been suggested to be more pathogenic than cattle C-BSE^9^. Konold et al. reported incomplete attack rates after intracranial inoculation of pigs with C-BSE^6^ and pigs are apparently resistant to C-BSE after oral challenge^4^. The transmissibility of chronic wasting disease (CWD) to the pig has also been demonstrated, and PrP^Sc^ accumulation can be detected in these animals by RT-QuIC as soon as 6 months after inoculation. However, pigs accumulated low levels of PrP^CWD^ and subsequent bioassays in PrP porcine transgenic mice demonstrated a strong transmission barrier to CWD prion propagation in pigs^10^.

PrP^Sc^ deposition and infectivity have been also reported in pigs inoculated with an isolate of classical scrapie, but these animals did not develop clinical signs of TSE and PrP^Sc^ could only be detected by conventional techniques in aged animals^11^. Propagation of other classical scrapie isolates in piglets or transgenic mice overexpressing porcine PrP^C^ (PoPrP-Tg001 mice, hereafter referred to as tgPo) has been unsuccessful^12,13^. Surprisingly, it was shown that one atypical scrapie isolate was able to propagate in tgPo mice.

Although a strong transmission barrier was observed, this atypical scrapie isolate experimented a drastic molecular shift, acquiring similar biochemical properties to those of SBSE when transmitted to tgPo mice^13^. However, the same atypical scrapie isolate was not able to reproduce the infection when inoculated again in tgPo mice in a posterior study^14^ leaving the question of whether pigs are susceptible to atypical scrapie still open. The emergence of C-BSE prions from atypical scrapie isolates was further demonstrated both in vivo and in vitro. Transmission of atypical scrapie isolates from 5 different European countries to mice expressing the bovine prion protein resulted in the propagation of C-BSE. Serial in vitro amplification of these atypical scrapie isolates by protein misfolding cyclic amplification (PMCA) in bovine and ovine PrP substrates demonstrated that C-BSE emerges as the dominant strain in both PrP substrates^15^.

In this study, we tested the susceptibility of pigs to two atypical scrapie isolates by intracerebral inoculation. Challenged animals did not show clinical signs compatible with a TSE, and collected tissues tested negative for PrP^Sc^ accumulation by conventional techniques. In addition, we did not find infectivity in brain tissues from atypical scrapie-inoculated pigs by mouse bioassay in tgPo mice. Surprisingly, PMCA reactions seeded with brain material from seven of the pigs resulted positive for the propagation of C-BSE prions. Positive PMCA reactions were obtained from pigs inoculated with either of the original atypical isolates. These results corroborate that C-BSE can emerge during passage of atypical scrapie between different livestock species and that pigs could act as a reservoir for C-BSE.

## Results

### No PrP^Sc^ accumulation is detected in brains from pigs inoculated with atypical scrapie

Atypical scrapie-inoculated pigs were monitored periodically by veterinary staff. None of the animals showed clinical signs compatible with a TSE. However, several pigs developed concomitant pathologies and were euthanized when their welfare was compromised. Other animals were electively euthanized to test the evolution of the disease (Table 1).

**Table 1:** Inoculation of pigs with atypical scrapie isolates.

ID	Isolate	Sex	Months post-inoculation^a^	Reasons for euthanasia
P-1217	PS152	Female	28	Elective
P-1216	PS152	Female	44	Elective
P-1213	PS152	Female	48	Stomach ulcer
P-1212	PS152	Female	50	Uterine infection
P-1215	PS152	Female	62	Elective
P-1214	PS152	Female	72	Elective
P-1226	TOA3	Female	22	Inner ear infection
P-1231	TOA3	Neutered male	35	Elective
P-1228	TOA3	Female	47	Uterine infection
P-1229	TOA3	Female	48	Uterine infection and intestinal haemorrhage
P-1227	TOA3	Female	62	Elective
P-1230	TOA3	Female	67	Uterine infection

^a^Pigs were euthanized without showing clinical signs of TSE.

The evaluation of the brain sections stained with hematoxylin–eosin did not reveal any spongiform changes. None of the challenged pigs showed PrP^Sc^ accumulation in the brain by enzyme immunoassay (EIA) (Table 2), immunohistochemistry or western blotting (not shown).

**Table 2:** Enzyme immunoassay test results for atypical scrapie inoculated pigs.

ID	Brain area	EIA result^a^
P-1212	Frontal cortex	0.018
	Cerebellum	0.019
P-1213	Frontal cortex	0.024
	Cerebellum	0.018
P-1214	Frontal cortex	0.020
	Cerebellum	0.019
P-1215	Frontal cortex	0.019
	Cerebellum	0.022
P-1216	Frontal cortex	0.026
	Cerebellum	0.020
P-1217	Frontal cortex	0.018
	Cerebellum	0.015
P-1226	Frontal cortex	0.021
	Cerebellum	0.016
P-1226	Frontal cortex	0.022
	Cerebellum	0.017
P-1228	Frontal cortex	0.029
	Cerebellum	0.027
P-1229	Frontal cortex	0.021
	Cerebellum	0.018
P-1230	Frontal cortex	0.029
	Cerebellum	0.026
P-1231	Frontal cortex	0.019
	Cerebellum	0.024
*Atypical scrapie clinical sheep*	Frontal cortex	2.878
	Cerebellum	0.825
*Sheep BSE inoculated pig*	Frontal cortex	3.372
	Cerebellum	3.374

^a^EIA test was performed using a negative cut off value of 0.14 absorbance units.

All pigs were also tested for infectivity in brain tissues by mouse bioassay. TgPo mice were selected as an appropriate model for the bioassay because this line overexpresses pig PrP (fourfold the level of expression in pig brain^13^), therefore representing no transmission barrier for porcine prions. TgPo mice were euthanized after 650 days post inoculation (dpi) without showing clinical signs compatible with a TSE. None of these mice were positive for PrP^Sc^ accumulation in their brains. Details of the mouse bioassay are represented in Table 3.

**Table 3:** Bioassay in tgPo mice.

Brain homogenates^a^	Attack rate	Survival time, dpi
P-1212	0/5	> 650
P-1213	0/5	> 650
P-1214	0/5	> 650
P-1215	0/7	> 650
P-1216	0/5	> 650
P-1217	0/5	> 650
P-1226	0/6	> 650
P-1227	0/6	> 650
P-1228	0/5	> 650
P-1229	0/5	> 650
P-1230	0/5	> 650
P-1231	0/5	> 650

^a^Mice were inoculated with a 10% brain pool from each atypical scrapie-challenged pig.

### C-BSE prions emerge during the in vitro propagation of brain isolates from pigs challenged with atypical scrapie

In vitro propagation by PMCA demonstrated recently that C-BSE prions can be present in ovine atypical scrapie isolates. C-BSE can also emerge during the inter-species passage of atypical scrapie^15^. To detect whether C-BSE emerged in the atypical scrapie-challenged pigs, brain material from these animals was subjected to PMCA using substrates from transgenic mice expressing bovine PrP (tgBov). This substrate was selected because it is the most permissive for C-BSE amplification, whereas several PMCA rounds are required to amplify C-BSE using tgPo brain as a substrate^14^.

Interestingly, PrP^Sc^ amplification was detected in reactions seeded with brain material from 7 pigs (Supplementary table 1). Each brain area was tested in quadruplicate and positive reactions were confirmed by retesting the original sample in 12 PMCA replicates.

**Figure 1:**
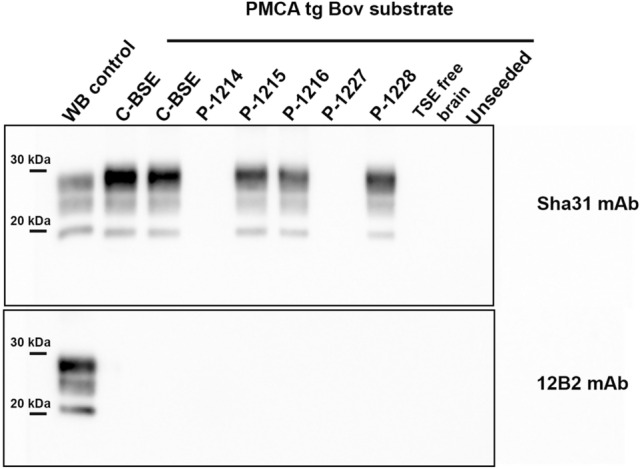
PrP^res^ detected in PMCA reactions seeded with brain material (parietal cortex) from atypical scrapie inoculated pigs. Positive PMCA reactions show an identical glycoform pattern to that of cattle C-BSE prions and/or cattle C-BSE propagated in tgBov substrate (third line). Positive reactions were obtained from pigs inoculated with both original atypical scrapie isolates (PS152 isolate: P-1215 and P-1216; TOA3 isolate: P-1228). PMCA reactions seeded with brain homogenate from a TSE negative sheep and unseeded PMCA reactions are included as controls of the technique. PMCA reactions were subjected to 3 amplification rounds (15 s sonication, 14 min 50 s incubation at 39 °C) in a Qsonica700 sonicator and analyzed for PrP^res^ by western blot using the monoclonal anti-PrP antibodies Sha31 and 12B2. WB control = classical scrapie isolate.

PrP^res^ western blot profile from positive PMCA reactions was characterized by a predominance of the di-glycosylated band, non-glycosylated band at ~ 19 kDa and lack of detection with the 12B2 antibody (Fig. 1). These features are undistinguishable from C-BSE prions and PMCA reaction products seeded with C-BSE prions. No PrP^res^ was detected in unseeded PMCA reactions or those seeded with brain homogenate from a TSE negative sheep (Fig. 1).

## Discussion

The outbreak of C-BSE was followed by the appearance of TSE in species that had never been diagnosed with prion diseases and the emergence in humans of vCJD^16–18^. However, no natural prion disease has been described in pigs, even though they were exposed to C-BSE contaminated feed^12^. Posterior experimental challenges in pigs and mice expressing porcine PrP have demonstrated that, although they are not completely resistant, pigs present a robust transmission barrier for C-BSE prions^4,14,19^.

However, the possible transmission of a TSE to swine is a public health concern due to the wide use of pork as a source of human food, and the increasing use of pigs as tissue donors, being reported a case of vCJD in a human patient receiving a swine dura mater graft^20^. Although pigs are apparently non-susceptible to C-BSE after oral challenge^4,5,21^, infectivity has been detected in tissues from pigs orally inoculated with classical scrapie or CWD^10,11^. In addition, these positive orally inoculated pigs are often subclinical, what could represent a public health concern, considering that these animals could reach the slaughterhouse without showing signs suggestive of prion disease.

In the present study, we evaluated the transmissibility of atypical scrapie to pigs. Pigs were euthanized between 22- and 72-months post inoculation (mpi), and their tissues tested for PrP^Sc^ accumulation and infectivity. We did not find evidence of transmission of atypical scrapie to any of the animals by EIA (Table 2), western blotting, or mouse bioassay (Table 3). PrP^Sc^ accumulation can be detected in BSE-challenged pigs at 34 mpi^4^, and at 22 mpi when inoculated with SBSE^7^. Although scrapie or CWD-inoculated pigs do not show clinical signs, PrP^Sc^ presence can be found in scrapie-challenged animals at 51 mpi^11^ and as early as 6 mpi in the case of CWD^10^.

Our main goal was to test the ability of atypical scrapie/Nor98 strain to propagate in swine, given that mice expressing porcine PrP (PoPrP-Tg001/tgPo mice) showed to be susceptible to atypical scrapie inoculation. One atypical scrapie isolate adapted to this transgenic line, reaching a 100% attack rate and rapid incubation periods in serial passages^13^, a similar adaptation to that observed with the C-BSE agent^19^. However, when this atypical scrapie isolate was tested for propagation in tgPo mice again, together with other atypical scrapie isolates, no positive results were obtained, in vitro nor in vivo^14^. These results, together with the negative transmissions showed in the present study, reinforce the conclusion that porcine species is highly resistant to atypical scrapie. However, we only performed one passage in tgPo mice, and further passages in this line and/or PMCA analysis of tgPo brains to detect any possible prion replication would be of interest.

However, it was demonstrated that C-BSE prions can be present as a minor variant in ovine atypical scrapie isolates and that C-BSE can emerge during the passage of these isolates to bovine PrP mice^15^. Considering that the aforementioned atypical scrapie isolate also acquired BSE-like properties when transmitted to tgPo mice^13^, and that C-BSE is the only prion that efficiently propagates in swine PrP^4,7,14^, we decided to investigate whether C-BSE prions could emerge from atypical scrapie during the ovine-porcine interspecies transmission.

Interestingly, PMCA reactions seeded with brain material from 7 pigs propagated in tgBov substrate showing PrP^res^ with identical biochemical characteristics to those of C-BSE (Fig. 1). Positive C-BSE amplification was detected in the brain of pigs inoculated with either the PS152 or TOA3 atypical scrapie isolates, at minimum incubation periods of 28- and 35-months post inoculation, respectively. From each animal, positive reactions were not obtained from all brain areas tested (Supplementary table 1). Although PrP^res^ amplified from the pigs showed C-BSE biochemical characteristics, further bioassays in tgBov mice are required to know whether these prions replicate the neuropathological features of C-BSE.

Altogether, our results and data obtained from transmission studies of prions to pigs, tgPo mice and in vitro studies using porcine substrate have shown that pig PrP has a very limited ability to sustain prion replication. No significant polymorphisms have been described for pig *PRNP*^22^, and it has been suggested that the conformational flexibility of pig PrP sequence is very low, limiting the number of PrP^Sc^ conformations able to produce misfolding^14^. No differences have been found between pig and minipig PrP sequences either^23^, suggesting that the conclusions obtained here could be extrapolated to domestic, non-experimental pigs. However, using tgBov substrate, we have demonstrated in vitro the presence of C-BSE seeding activity in some pig brain areas, suggesting that C-BSE prions emerged during the transmission of ovine atypical scrapie prions to pigs. Interestingly, C-BSE prions did not emerge from brain material of all the pigs, and, of those from which it did emerge, it was not detected in all brain areas tested. No correlation between time after inoculation and BSE emergence was found either. When the emergence of C-BSE from atypical scrapie in PMCA was described, it was associated to low levels of C-BSE prions that were present in the original atypical scrapie isolates^15^. It is possible that this result is related to the great resistance that pigs present to prion diseases, making the penetrance of the BSE prions that could be present in the original inoculum incomplete. In addition, considering that the amount of C-BSE conformers in the atypical scrapie inocula is probably very reduced and perhaps not homogeneously distributed throughout the isolate, it is also possible that not all the pigs received a sufficient amount of C-BSE conformers capable of being detected by PMCA. Finally, we should consider that PMCA amplification of prions is sometimes a stochastic phenomenon, which could explain why no C-BSE propagation was obtained from some of the pigs. It could be also discussed that C-BSE emergence from the pig brains could be related to persistence of the original atypical scrapie inoculum. However, C-BSE amplification was not obtained from all of the pigs and, in some of them (i.e. P-1217 and P-1231) C-BSE propagation was detected in caudal regions of the brain (cerebellum or occipital cortex) but not in more rostral areas (such as parietal cortex). If C-BSE amplification from pig brain samples were associated to inoculum persistence and not bona fide propagation of C-BSE prions it would be expected that such amplification would be detected mainly in the most rostral areas of the brain. Finally, even though the titer generated was not enough to produce disease in the pigs, these results evidence again the issue that pigs could act as subclinical reservoirs for prion diseases as observed with scrapie and CWD, and that the presence of prions can be detected in pigs short after exposure to prions^7,10,11^.

In conclusion, our findings suggest that, although pigs present a strong transmission barrier against the propagation of atypical scrapie, they can propagate low levels of C-BSE prions. The prevalence of atypical scrapie and the presence of infectivity in tissues from atypical scrapie infected sheep are underestimated^24,25^. Given that pigs have demonstrated being susceptible to other prion diseases, and to propagate prions without showing signs of disease, the measures implemented to ban the inclusion of ruminant proteins in livestock feed must not be interrupted.

## Material and methods

### Ethics statement

All animal experiments were performed in strict accordance with the European Community Council Directive 2010/63/EU for the protection of animals used for experimental and other scientific purposes. Experimental procedures in pigs were approved by the Ethics Committee of the University of Zaragoza (Permit number: PI 13/10) and were carried out in strict accordance with the Spanish Policy on Animal Protection (RD 53/2013). The CISA-INIA Ethics Committee approved the experimental protocol in mice (Permit number: CEEA 2009/004). All experimental procedures in animals comply with the ARRIVE guidelines.

### Atypical scrapie inocula

In the present study, two different atypical scrapie sources were used: PS152 isolate and TOA3 isolate.

PS152 isolate (French isolate) was derived from the brain of an AfRQ/AfRQ clinical sheep naturally affected by atypical scrapie. This isolate was provided by UMR INRA ENVT 1225, Interactions Hôtes Agents Pathogènes, École Nationale Vétérinaire de Toulouse.

TOA3 isolate (Catalan isolate) was obtained from the brain of an AfRQ/AfRQ clinical sheep naturally affected by atypical scrapie and provided by the prion diseases research group of the IRTA-CReSA and Universitat Autònoma de Barcelona.

### Experimental challenges in pigs

Twelve 9-month-old Sach miniature minipigs (11 females and 1 neutered male) were intracerebrally inoculated under general anesthesia. Animals were purchased from the Centro de Transferencia Tecnológica “La Chimenea”, Aranjuez, Madrid, Spain, which is part of the Instituto Madrileño de Investigación y Desarrollo Rural, Agrario y Alimentario (IMIDRA). Pigs were housed together in the BSL3 facility from Centro de Encefalopatías y Enfermedades Transmisibles Emergentes of the University of Zaragoza. The number of animals used was considered adequate to ensure both statistical significance and animal welfare in relation to housing. Females were selected mainly because they show better behavior and a lower tendency to aggression against other pigs.

Two groups of six animals were intracerebrally challenged with 0.5 ml of the PS152 isolate or the TOA3 isolate, respectively. Atypical scrapie inocula were prepared as 10% brain homogenate (w/v) in sterile saline solution. Each animal received 0.05 g of brain tissue under xylazine and ketamine anesthesia. The trephine was done using a dental drill. Inocula were administered using a 25G needle. Intracerebral injections were performed as previously described for prion transmission studies in pigs^4^. Briefly, each pig was inoculated into the frontal cortex of the left cerebral hemisphere. Before inserting the trephine, a parasagittal skin incision of 2 cm was made over the inoculation point to expose the periosteum. The injection was performed 1 cm lateral to the midline in the frontal region equidistant from the lateral canthus of the eye and the base of the cranial border of the pinna. After the inoculation, the incision was sutured, and pigs received intramuscular (IM) antibiotic treatment (procaine benzylpenicillin, 10 mg/kg) and flunixin (IM, 2.2 mg/kg) to achieve analgesia. Details of the bioassay are presented in the results section (Table 1).

After the intracerebral injection, pigs were monitored daily by animal husbandry staff. The possible appearance of locomotor impairments was monitored three days per week. Veterinary clinical assessments were performed monthly in order to detect any clinical sign compatible with a prion disease, which in this species include behavioral alterations, depression, hyperreaction to stimuli, abnormal positions of the head and ears, confusion and ataxia^7^.

### Necropsy and tissue sample collection

Animals were sacrificed by intravenous pentobarbital injection (DOLETHAL; 10 mg/kg) followed by exsanguination. Pigs were euthanized between 632- and 2154-days post-inoculation without showing clinical signs of prion disease. Several pigs were euthanized due to the development of conditions nonrelated to prion disease that compromised their welfare. Due to the great cost involved in maintaining these animals, the rest of the pigs were electively slaughtered when a minimum of two years had passed after inoculation. PrP^Sc^ accumulation in the brain was evaluated after every necropsy. Elective euthanasia of the animals was carried out at similar post-inoculation times for both inoculation groups except for pig P-1216, which was euthanized a few months after P-1231 because the latter showed no presence of PrP^Sc^ in the brain, and it was decided to postpone the euthanasia of the next animal.

Necropsies were conducted systematically, and tissue samples were collected from the central nervous system using sterile prion-free material and equipment.

All samples were collected in duplicate, one sample was fixed in 10% formalin for the histological analysis and the other sample was stored at − 80 °C for further biochemical assays.

### Enzyme immunoassay (EIA) for the detection of PrP^Sc^

Collected tissues (300 mg per sample) were analyzed by a ligand-based enzyme immunoassay (IDEXX HerdChek® BSE-Scrapie Antigen Test, referred to as EIA) as previously described^7,26^. EIA test was performed following the manufacturer´s instructions, using the conjugate indicated for cattle and applying a negative cut-off value of 0.14 absorbance units.

Frontal cortex and cerebellum were selected as areas for the possible detection of atypical scrapie prions.

### Histopathology and immunohistochemistry

Tissue samples were fixed in formalin for 48 h and then embedded in paraffin. 4 µm-thick sections were cut using a microtome and mounted on glass slides. Some sections were stained with hematoxylin and eosin for the histopathological study. PrP^Sc^ detection was performed by immunohistochemistry using the monoclonal primary antibody 2G11 (1:400; Bertin Bioreagent) as previously described^7,27^.

### Mouse bioassay

PoPrP-Tg001 mouse line (tgPo) was used in the bioassay experiments. This mouse line expresses the porcine PrP^C^ (around fourfold the level of expression in pig brain) under the control of the mouse *prnp* gene promoter in a mouse PrP^0/0^ background^19^. Bioassays were performed as reported previously in experimental procedures from our group^28^. Briefly, all inocula were prepared from brain tissues as 10% (w/v) homogenates in 5% glucose. Individually identified 6–10 week-old mice were anesthetized with isoflurane and inoculated with 2 mg of brain homogenate in the right parietal lobe using a 25-gauge disposable hypodermic needle. Mice were observed daily, and the neurological status was assessed weekly. When mice became too aged (> 650 dpi), animals were euthanized because of ethical reasons. Once euthanized, necropsy was performed, and the brain was collected. A part of the brain was fixed by immersion in 10% formalin to quantify spongiform degeneration by histopathology and proteinase K (PK) resistant PrP accumulation (PrP^res^) by immunohistochemistry (IHC) or histoblotting and the other part was frozen at − 20 °C to determine the presence of PrP^res^ by western blot.

### PMCA amplification

Brains from transgenic mice overexpressing bovine PrP (tgBov)^29^ were used to prepare the PMCA substrate (10% wt/vol in PMCA conversion buffer) which was supplemented with 0.25% wt/vol of low molecular weight dextran. The PMCA technique was carried out similarly to that previously described. PMCA reactions (50 μl final volume) were performed in 96 well microplates and subjected to 3 amplification rounds (15 s sonication, 14 min 50 s incubation at 39 °C) in a Qsonica700 sonicator. These conditions ensure a positive amplification of 10^–9^ diluted reference C-BSE isolate. After each round, reaction products were diluted 1/10 in fresh substrate and subjected to a new PMCA round. Pig brain regions for PMCA amplification were selected according to the distribution of neuropathological lesions reported in pigs inoculated with C-BSE and sheep BSE^7,21^. In these animals, most severe lesions are described in areas such as cerebral and cerebellar cortices, white matter structures and midbrain. Frontal cortex was not subjected to PMCA since pigs were inoculated in this brain area and a certain level of inoculum persistence could result in positive amplification by an ultrasensitive technique such as PMCA^30^.

Each pig brain area was tested in quadruplicate and the PMCA reaction products were blindly analyzed by western blot for the presence of PrP^res^. In order to detect any possible cross contamination that could lead to a false positive result, unseeded controls were placed in the microplate every 8 seeded wells and the complete plate was discarded when non-specific amplification was detected. In case of positive amplification, 12 replicates of the sample were retested under the same conditions. When all brain areas from an animal were negative for PMCA amplification, one selected brain area was retested in 12 replicates to confirm the negative result.

### Western blotting

Biochemical analysis of PrP^res^ from PMCA products was performed by western blotting as previously described^15^. Fresh samples were treated with PK (50 μg/ml), subjected to electrophoresis and blotted onto membranes using the Prionics AG Check® Western BSE test protocol. Immunodetection was performed using Sha31 (1 µg/ml)^31^ and 12B2 (4 µg/ml)^32^ monoclonal antibodies.

## Supplementary Information

Supplementary Information.

## Data Availability

Data available within the article and its supplementary materials.

## References

[1] Prusiner SB (1991). Molecular biology of prion diseases. Science.

[2] Wilesmith JW, Wells GA, Cranwell MP, Ryan JB (1988). Bovine spongiform encephalopathy: epidemiological studies. Vet. Rec..

[3] Wilesmith JW, Ryan JB, Atkinson MJ (1991). Bovine spongiform encephalopathy: epidemiological studies on the origin. Vet. Rec..

[4] Wells GA, Hawkins SA, Austin AR, Ryder SJ, Done SH, Green RB, Dexter I, Dawson M, Kimberlin RH (2003). Studies of the transmissibility of the agent of bovine spongiform encephalopathy to pigs. J. Gen. Virol..

[5] Dawson M, Wells GA, Parker BN, Scott AC (1990). Primary parenteral transmission of bovine spongiform encephalopathy to the pig. Vet. Rec..

[6] Konold T, Spiropoulos J, Chaplin MJ, Thorne L, Spencer YI, Wells GA, Hawkins SA (2009). Transmissibility studies of vacuolar changes in the rostral colliculus of pigs. BMC Vet. Res..

[7] Hedman C, Bolea R, Marin B, Cobriere F, Filali H, Vazquez F, Pitarch JL, Vargas A, Acin C, Moreno B, Pumarola M, Andreoletti O, Badiola JJ (2016). Transmission of sheep-bovine spongiform encephalopathy to pigs. Vet. Res..

[8] Hedman C, Otero A, Douet JY, Lacroux C, Lugan S, Filali H, Corbiere F, Aron N, Badiola JJ, Andreoletti O, Bolea R (2018). Detection of PrPres in peripheral tissue in pigs with clinical disease induced by intracerebral challenge with sheep-passaged bovine spongiform encephalopathy agent. PLoS One.

[9] Padilla D, Beringue V, Espinosa JC, Andreoletti O, Jaumain E, Reine F, Herzog L, Gutierrez-Adan A, Pintado B, Laude H, Torres JM (2011). Sheep and goat BSE propagate more efficiently than cattle BSE in human PrP transgenic mice. PLoS Pathog..

[10] Moore SJ, West Greenlee MH, Kondru N, Manne S, Smith JD, Kunkle RA, Kanthasamy A, Greenlee JJ (2017). Experimental transmission of the chronic wasting disease agent to swine after oral or intracranial inoculation. J. Virol..

[11] Greenlee JJ, Kunkle R, Smith JD, West GM (2016). Scrapie in swine: a diagnostic challenge. Food Saf..

[12] Matthews D, Cooke BC (2003). The potential for transmissible spongiform encephalopathies in non-ruminant livestock and fish. Rev. Sci. Technol..

[13] Espinosa JC, Herva ME, Andreoletti O, Padilla D, Lacroux C, Cassard H, Lantier I, Castilla J, Torres JM (2009). Transgenic mice epressing porcine prion protein are resistant to cassical srapie but susceptible to sheep bovine spongiform encephalopathy and atypical scrapie. Emerg. Infect. Dis..

[14] Espinosa JC, Marin-Moreno A, Aguilar-Calvo P, Benestad SL, Andreoletti O, Torres JM (2020). Porcine prion protein as a paradigm of limited susceptibility to prion strain propagation. J. Infect. Dis..

[15] Huor A, Espinosa JC, Vidal E, Cassard H, Douet JY, Lugan S, Aron N, Marin-Moreno A, Lorenzo P, Aguilar-Calvo P, Badiola J, Bolea R, Pumarola M, Benestad SL, Orge L, Thackray AM, Bujdoso R, Torres JM, Andreoletti O (2019). The emergence of classical BSE from atypical/Nor98 scrapie. Proc. Natl. Acad. Sci. U S A.

[16] Hill AF, Desbruslais M, Joiner S, Sidle KC, Gowland I, Collinge J, Doey LJ, Lantos P (1997). The same prion strain causes vCJD and BSE. Nature.

[17] Bruce ME, Will RG, Ironside JW, McConnell I, Drummond D, Suttie A, McCardle L, Chree A, Hope J, Birkett C, Cousens S, Fraser H, Bostock CJ (1997). Transmissions to mice indicate that 'new variant' CJD is caused by the BSE agent. Nature.

[18] Sigurdson CJ, Miller MW (2003). Other animal prion diseases. Br. Med. Bull..

[19] Castilla J, Gutierrez-Adan A, Brun A, Doyle D, Pintado B, Ramirez MA, Salguero FJ, Parra B, San Segundo FD, Sanchez-Vizcaino JM, Rogers M, Torres JM (2004). Subclinical bovine spongiform encephalopathy infection in transgenic mice expressing porcine prion protein. J. Neurosci..

[20] Heath CA, Barker RA, Esmonde TF, Harvey P, Roberts R, Trend P, Head MW, Smith C, Bell JE, Ironside JW, Will RG, Knight RS (2006). Dura mater-associated Creutzfeldt-Jakob disease: experience from surveillance in the UK. J. Neurol. Neurosurg. Psychiatry.

[21] Ryder SJ, Hawkins SA, Dawson M, Wells GA (2000). The neuropathology of experimental bovine spongiform encephalopathy in the pig. J. Comp. Pathol..

[22] Martin T, Hughes S, Hughes K, Dawson M (1995). Direct sequencing of PCR amplified pig PrP genes. Biochim. Biophys. Acta.

[23] Meng L, Zhao D, Liu H, Yang J, Ning Z (2005). Single nucleotide polymorphisms of the prion protein gene (PRNP) in Chinese pig breeds. Xenotransplantation.

[24] Andreoletti O, Orge L, Benestad SL, Beringue V, Litaise C, Simon S, Le Dur A, Laude H, Simmons H, Lugan S, Corbiere F, Costes P, Morel N, Schelcher F, Lacroux C (2011). Atypical/Nor98 scrapie infectivity in sheep peripheral tissues. PLoS Pathog..

[25] Ru G (2017). Do we need to explain the occurrence of atypical scrapie?. Vet. Rec..

[26] Garza MC, Monzon M, Marin B, Badiola JJ, Monleon E (2014). Distribution of peripheral PrP(Sc) in sheep with naturally acquired scrapie. PLoS One.

[27] Andreoletti O, Berthon P, Marc D, Sarradin P, Grosclaude J, van Keulen L, Schelcher F, Elsen JM, Lantier F (2000). Early accumulation of PrP(Sc) in gut-associated lymphoid and nervous tissues of susceptible sheep from a Romanov flock with natural scrapie. J. Gen. Virol..

[28] Torres JM, Espinosa JC, Aguilar-Calvo P, Herva ME, Relano-Gines A, Villa-Diaz A, Morales M, Parra B, Alamillo E, Brun A, Castilla J, Molina S, Hawkins SA, Andreoletti O (2014). Elements modulating the prion species barrier and its passage consequences. PLoS One.

[29] Castilla J, Adan AG, Brun A, Pintado B, Ramirez MA, Parra B, Doyle D, Rogers M, Salguero FJ, Sanchez C, Sanchez-Vizcaino JM, Torres JM (2003). Early detection of PRPres in BSE-infected bovine PrP transgenic mice. Arch. Virol..

[30] Martin D, Reine F, Herzog L, Igel-Egalon A, Aron N, Michel C, Moudjou M, Fichet G, Quadrio I, Perret-Liaudet A, Andreoletti O, Rezaei H, Beringue V (2021). Prion potentiation after life-long dormancy in mice devoid of PrP. Brain Commun..

[31] Feraudet C, Morel N, Simon S, Volland H, Frobert Y, Creminon C, Vilette D, Lehmann S, Grassi J (2005). Screening of 145 anti-PrP monoclonal antibodies for their capacity to inhibit PrPSc replication in infected cells. J. Biol. Chem..

[32] Langeveld JP, Jacobs JG, Erkens JH, Bossers A, van Zijderveld FG, van Keulen LJ (2006). Rapid and discriminatory diagnosis of scrapie and BSE in retro-pharyngeal lymph nodes of sheep. BMC Vet. Res..

