# Supplementary material for: Classical BSE prions emerge from asymptomatic pigs challenged with atypical/Nor98 scrapie
Source: Sci Rep. 2021 Aug 31;11:17428. doi: 10.1038/s41598-021-96818-2 (PMC8408226; doi:10.1038/s41598-021-96818-2)

Supplementary table 1: Protein Misfolding Cyclic Amplification of pig brain samples using brains from tgBov as substrate

ID	Brain area[a]	PMCA positive reactions (tgBov substrate) 1st testing	PMCA positive reactions (tgBov substrate) 2nd testing
P-1212	PC	0/4	
	OC	0/4	
	CBL	0/4	
	MES	0/4	0/12
	CSC	0/4	
P-1213	PC	0/4	
	OC	0/4	
	CBL	0/4	
	MES	0/4	0/12
	CSC	0/4	
P-1214	PC	0/4	
	OC	0/4	
	CBL	0/4	
	MES	0/4	0/12
	CSC	0/4	
P1215	PC	**4/4**	**10/12**
	OC	**2/4**	**4/12**
	CBL	**1/4**	**ND**
	MES	0/4	

	CSC	0/4	
P1216	PC	**2/4**	**2/12**
	OC	0/4	
	CBL	0/4	
	MES	0/4	
	CSC	0/4	
P1217	PC	0/4	
	OC	0/4	
	CBL	**1/4**	**4/12**
	MES	**1/4**	**5/12**
	CSC	0/4	
P1226	PC	0/4	0/12
	OC	0/4	
	CBL	0/4	
	MES	0/4	
	CSC	0/4	
P1227	PC	0/4	0/12
	OC	0/4	
	CBL	0/4	
	MES	0/4	
	CSC	0/4	
P1228	PC	**2/4**	**4/12**
	OC	0/4	
	CBL	0/4	
	MES	0/4	

	CSC	0/4	
P1229	PC	0/4	
	OC	0/4	
	CBL	0/4	
	MES	0/4	
	CSC	**2/4**	**2/12**
P1230	PC	**1/4**	**ND**
	OC	0/4	
	CBL	**3/4**	**4/12**
	MES	**1/4**	**ND**
	CSC	0/4	
P1231	PC	0/4	
	OC	**1/4**	**2/12**
	CBL	**2/4**	**7/12**
	MES	**1/4**	**7/12**
	CSC	0/4	

[a]PC : Parietal cortex ; OC : Occipital cortex ; CBL : Cerebellum ; MES : Mesencephalon ; CSC : Cervical spinal cord.

Full-length western blots corresponding to the main figure in the text are provided on the following pages. Different exposure times are displayed.

Figure 1 : Top panel (Sha31 antibody)

5s:

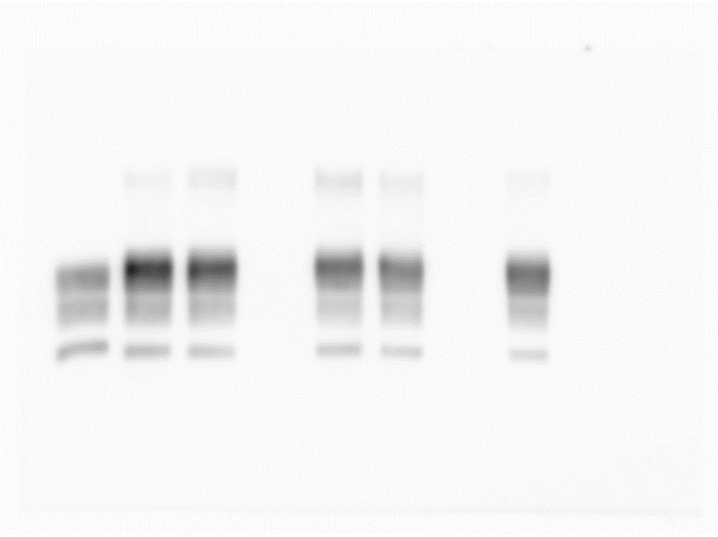

10s:

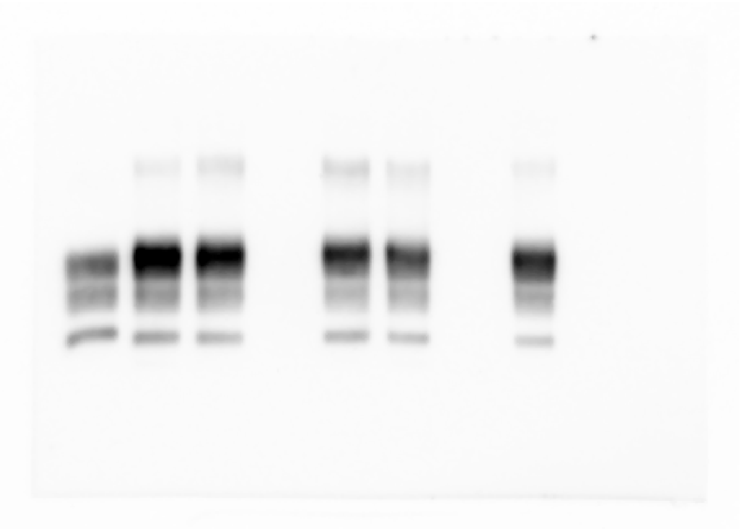

20s:

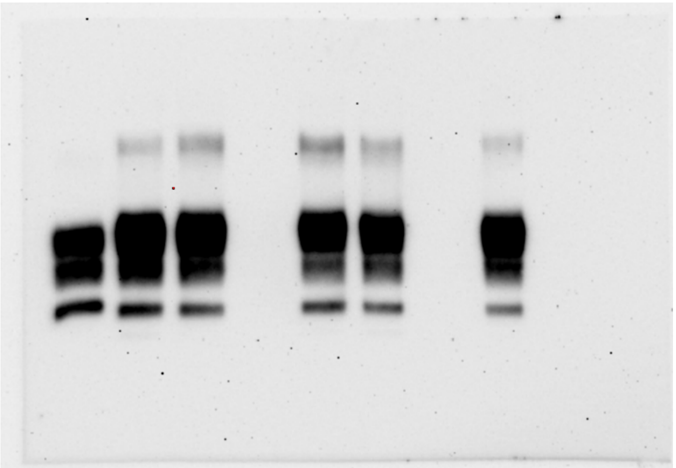

Figure 1 : Low panel (12B2 antibody)

5s:

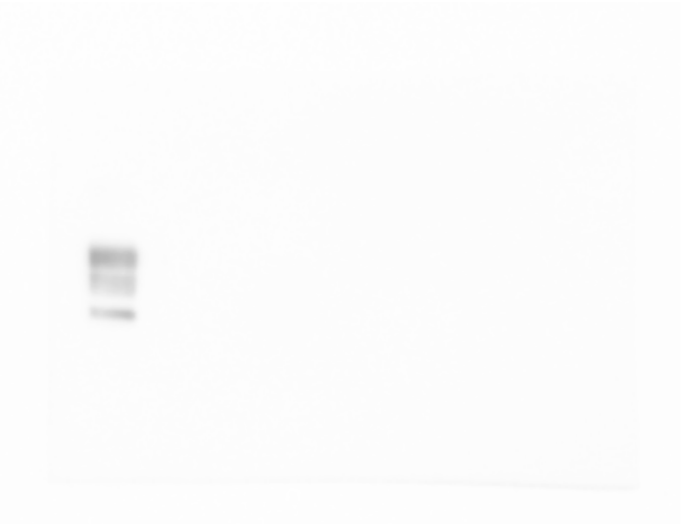

10s:

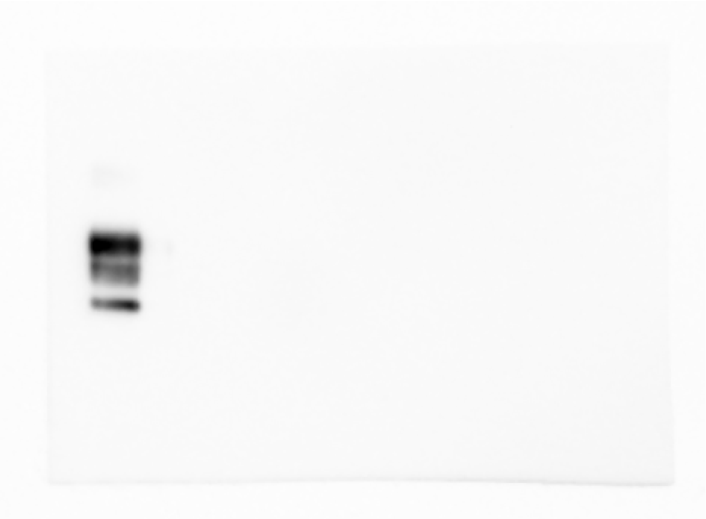

20s:

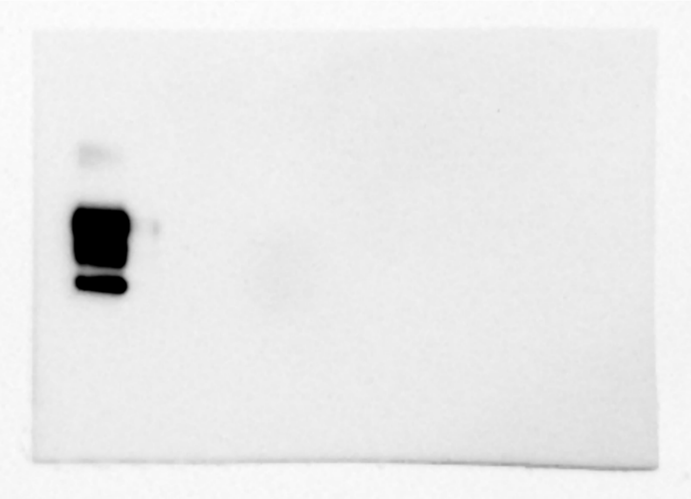

Supplement: Supplementary file 1 — Supplementary Information. [file 41598_2021_96818_MOESM1_ESM.pdf]
